# Global surveillance, travel, and trade during a pandemic

**DOI:** 10.3906/sag-2004-175

**Published:** 2020-04-21

**Authors:** Ceren ÇETİN, Ateş KARA

**Affiliations:** 1 Department of Pediatric Infectious Diseases, Kartal Lütfi Kırdar Research Hospital, University of Medical Sciences, İstanbul Turkey; 2 Department of Pediatrics, Pediatric Infectious Diseases, Faculty of Medicine, Hacettepe University, Ankara Turkey

**Keywords:** Global surveillance, travel, trade, pandemic

## Abstract

Pandemics have had very important consequences in human history. Lots of people lost their lives and countries have been intensively affected in terms of socioeconomic problems. Unfortunately, avoidance of pandemics and limiting the spread are still currently not always possible. Maybe the most important factor for this is the increasing frequency of traveling. Increasing airline traveling rate also increases the rate of spread. Global organizations like the World Health Organization and United Nations are trying to play a supreme role over the countries. Pandemics do not have borders; therefore, efforts should be given globally, definition of pandemic should be established as soon as possible, and protective measures should be shared with countries. If these are not done, severe health consequences and serious economic problems are inevitable.

## 1. Introduction

Pandemic is defined as an epidemic occurring worldwide or over a very wide area that crosses international borders and affects a great number of people [1]. According to the World Health Organization (WHO), a pandemic is considered to have begun once a disease not encountered before in a population emerges and once the agent of the disease spreads easily and constantly among people and causes a virulent disease [2]. The fact that a disease is prevalent and leads to the loss of a large number of people does not make it sufficient to be defined as a pandemic, it should also be transmissible [2]. A pandemic increases morbidity and mortality in a very large geographic area and provokes social and political deterioration. It has a vital position throughout human history and in the development of civilizations. Due to profound socioeconomic problems caused by pandemics, they have resulted in changes in the agenda of countries and put forward issues, most particularly sanitation rules, that people should pay attention to while coexisting. 

## 2. Epidemics and pandemics

Throughout history, there have been many epidemics like variola and tuberculosis. One of the most destructive pandemics was the plague that killed an estimated 75–200 million people in the 14th century. Considering that the world population was approximately 475 million in that century, the fatal effect of the pandemic can be comprehended more easily [3]. 

The plague had begun in China and Central Asia and spread to the whole world. The transmission of plague to Europe became possible by the Chinese merchandise sold to Europe by Asian traders [4]. It is known that the fleas and mice on the ship were effective in the spread of the disease. In the meantime, Crimean Tatars had invaded Genoese harbor and thrown their dead bodies carrying the plague using catapults into the city and transmitted the disease to the Italians [4]. The first Italian cities to encounter the plague were Genoa, Messina, and Venice. Afterwards, the plague arrived to Paris in 1348 and affected London in 1349, and after having penetrated into Scotland and Scandinavia, it reached to its origin, the land of the Tatars. It caused death of one-thirds of people living in Europe. Shortly after the spread of the pandemic, people experienced psychological destruction. Nonetheless, the aftermath of the plague saw a rise in the lack of confidence towards administrations, cutback in social activities and communication and led people to distance themselves from one another and caused an increase in product and labor. An economic collapse was seen in the world, mainly in Europe that had just started to recover from famine [4]. 

Millions of people were killed due to cholera pandemic in the 19th century [5]. There were four influenza pandemics in the 20th and 21st centuries. In 1918, the Spanish flu led to the death of 20–50 million people worldwide (world population was approximately 1.8 billion during that time) and estimated case-fatality rate was determined as 2–3% [6]. The 1957–58 pandemic, known as the Asian flu, resulted in the death of 1–4 million people worldwide, and the 1968-69 pandemic, referred to as the Hong Kong flu, caused the death of 1–4 million people worldwide. Estimated case-fatality rate of the 2009–2010 Influenza A (H1N1) pandemic was established as 0.02%. The age group the pandemic affected the most was children and young adults. It resulted in the death of 100–400 thousand people worldwide [6]. 

Last year around the end of 2019, a novel coronavirus pneumonia case group was identified in the Wuhan city of the province of Hubei in China. Rapidly spreading around the country, an epidemic arose and increasing number of cases started to be seen in other countries. In February 2020, the World Health Organization defined COVID-19, which meant 2019 coronavirus disease. The virus causing COVID-19 was referred to as severe acute respiratory syndrome coronavirus 2 (SARS-CoV-2). An interim guideline was published by the World Health Organization and the Centers for Disease Control and Prevention (CDC) [7,8]. COVID-19 was announced as a pandemic by the World Health Organization in 11 March 2020, and the pandemic is still continuing. 

## 3. Risk assessments

During a pandemic, steps include controlling and surveilling the outbreak, finding the center, bringing the source under control, preventing human-to-human transmission, ensuring social distancing, and determining vaccination and treatment agents [9]. 

In a model based on the 1918 flu pandemic analysis, it is assumed that one-third of the transmissions occurred in houses, one-third occurred in schools and workplaces, and the other one-third among the general public in the United States of America. Thus, an important control strategy would target the closing of schools and workplaces [10]. It should be seriously questioned whether hospitals would have the extreme capacity to treat a large number of patients or not. If not at sufficient capacity, necessary planning should be made by local supporters and public health officials in order to meet the deficit. Other measures include planning healthcare services in alternative settings other than hospitals, taking necessary infection control measures, and drawing up clear guidelines in favor of public health for patient consent [11]. It is necessary to form appropriate infrastructure and credible communication systems to coordinate public health intervention and plan and appoint leaders at national and local levels [12]. 

The World Health Organization (WHO) recommends countries to make their own pandemic risk evaluations since incidence of human and animal cases in different countries in the world and the procedures to be done thereafter would differ. WHO updated its Pandemic Influenza Risk Management Guideline in 2017. This guideline can be used to inform and conform national and international pandemic preparations and interventions. Countries should review and/or update their national flu preparation and intervention plans in order to reflect the approach taken in this guideline. Moreover, roles and responsibilities of WHO regarding pandemic preparations were also stated in terms of supporting member countries in line with the crisis and risk management policies. This guideline does not intend to replace national plans that should be developed by each country. National pandemic influenza risk evaluation aims at determining the probability and outcomes of events affecting public health at a global, national, and local level. It constitutes the basis to act against and decrease the negative consequences of public health risks [13]. It is attempted to make predictions with statistical models on when the agent would penetrate into the country or cities in the event of a pandemic [14]. 

Pandemic influenza phases reflect risk assessment of the global status concerning each and every influenza virus with a potential to be pandemic and to infect people. Initially, these assessments are carried out when these types of viruses are defined and afterwards updated according to virologic, epidemiologic, and clinical data obtained. 

Global phase represents the spread of the novel flu subtype to the world taking the disease it causes into consideration and separating it into interpandemic, alarm, pandemic, and transition phases. Global phases are used by WHO to convey global status. Pandemic phase is the period of global spreading. Transitions between interpandemic, alarm, and pandemic phases can be rapid and gradual [13]. 

As pandemic viruses emerge, countries and regions face different risks at different times. Therefore, it is strongly recommended for countries to develop their own national risk assessments by considering information provided by WHO. Hence, decisions for national risk management of any country are expected to be based on local risk assessments but also to be informed by global risk assessments [13]. WHO should be able to follow the development process of the pandemic and guide countries with appropriate methods like strengthened surveillance and active monitorization. WHO should make suggestions in medical and nonmedical issues for this purpose. WHO should announce data independently and in a transparent manner; thus, WHO requires accurate and rapid data flow from every government in order to fulfill its obligation [15]. United Nations (UN), on the other hand, is a much larger system. UN aids in countries to adapt themselves to necessary arrangements strengthening technical capacities of the countries during pandemics through the Peacekeeping Commission, United Nations Development Program, and United Nations Children’s Fund [16]. 

Risk assessment regarding pandemic influenza includes defining influenza viruses, reviewing significant virologic and clinical information on each and every influenza virus and classifying these as regards pandemic potency and probable outcomes. Evaluation of exposure aims at identifying exposure to an upsetting flu virus, individual groups with a probability to become ill, and defining the sensitivity of these groups in terms of immunity and disease severity. This process contains epidemiologic and sensitivity factors such as travel history, incubation period, and estimated transmission potential. Following risk and exposure assessments, these two assessments are finalized with a content evaluation. Content evaluation is the assessment of the setting the event has taken or is taking place. Content evaluation includes social, technological, scientific, economic, ethical, and political factors. Exposure and content evaluations are made, and risk is characterized. Risk characterization seeks to regulate the probability and impact of every risk. Risk characterization uses these evaluations in order to assess if a specific flu virus has the potential to become pandemic and to what extent the society will be affected by such event and thus to judge the urgency and scope of risk management activities. Risk assessment is a constant process during risk management continuity. For the evaluation of pandemic severity to be beneficial, it should be performed when public health decisions are required [13].

## 4. Measures, trade, and travel

To that end, surplus information should be provided in order to answer all critical questions regarding the pandemic that has emerged. Questions on new cases and their progression and those regarding the type of diseases and complications encountered, which group of patients would be severely ill and die (i.e. age groups and groups at risk for severe outcomes), whether or not the virus is susceptible to antiviral agents, the number of people that would become ill, and the impact of the cases on the use of healthcare services and workforce should be answered. These questions will be of help in directing decisions on vaccination production and utilization strategy, antiviral use, mobilization of healthcare sources, school closings, and social distancing strategies. 

Data responding to each critical question will be addressed in the context of three indicators. Each of these indicators will contain information obtained from various data types including virologic, epidemiologic, and clinical data. Data will be grouped so that they would be more accessible and comprehensible by the public and policy-makers. National action plans have been charted according to six categories of principal components of emergency risk management for health. These include policy and resource management; planning and coordination; information and information management; health infrastructure and logistics, healthcare and related services; and community emergency situation risk management capacities [13]. Research projects, their budget and the number of researchers to work on these projects should also be carefully constructed. Cooperation with the government for these plans is crucial [17]. 

Pandemics require globally compatible actions. Pandemics are extremely destructive events that can cause serious social, economic, and political stress. Preparation demands the approach of all communities in order to enable the world to respond quickly and effectively to decrease morbidity and mortality in the next pandemic. Not only the healthcare sector, but also all other sectors, individuals, families, and communities play a role in lightening the effects of a pandemic. Nonpharmaceutical interventions can be the only effective measure in many countries. During the onset of a pandemic, there would probably be no pandemic vaccination that would be effective against the novel virus. Non-pharmaceutical interventions in the early stage of the pandemic should be implemented in order to slow down transmission and decrease its effect. These interventions include social distancing (staying home when ill), coughing etiquette (covering the mouth with a handkerchief while coughing or sneezing), and hygiene rules such as washing the hands and cleaning the surfaces and objects touched. Extreme measures can be taken and implemented during severe pandemics including patients wearing masks (surgical masks), school closings, and diminishing contact between people. Nonpharmaceutical interventions will help decrease the number of people exposed to and afterwards infected by the virus [6]. 

Since pandemics necessitate an approach for the whole community, individuals and communities should be cared about, listened to, and relieved of anxieties. People should be informed on how to protect themselves and stop the spreading of the virus [18]. 

At the present time, airports, harbors and ports, road transportation and entry points to the country may have a critical role in the international transmission the diseases via persons, goods, and vehicles. Therefore, countries should be ready to detect and respond to any healthcare event that would cause international concern by bringing healthcare-based restrictions in international travel and trade. Thus, developing necessary public health capacities in entry points of the country will limit the spread of public health hazards (6). Particularly frequent travelers may have a part in accelerating the international spread of the virus during the early period of the pandemic. Hence, in the event of the onset of the pandemic being at a countryside where international travel is scarce, the transmission of the agent to frequent travelers will be much late and the spread of the pandemic will be much slower [19]. 

International Health Regulations (2005) try to “limit public health measures preventing unnecessary intervention to international travel and trade”. In order to reach this goal, WHO regularly makes recommendations on trade and travel measures regarding public health events. Along with not interfering with measures related to specific trade and travel, International Health Regulations (2005) mandate countries to inform WHO on the justifications of the time of interventions and important measures taken by the administration of the countries. This is defined as the cause of a more than 24-h delay in the movement of international passengers, luggage, cargos, containers, vehicles, and items by the International Health Regulations (2005). Apart from providing information to countries on these measures, WHO may demand from the implementing country to reevaluate these applications [13]. 

World Health Organization continues to make recommendations against travel and trade restrictions for countries fighting with the COVID-19 pandemic. Travel measures interfering significantly with international traffic can only be justified at the onset of a pandemic since countries may be allowed to rapidly implement preliminary measures even for a few days. These kinds of restrictions should be based on meticulous risk assessment, be proportional to public health risk, not last long, and be reevaluated regularly as the event advances. Travel ban to affected regions or rejection of entry of passengers traveling from affected regions is not effective in preventing case imports but may have important economic and social impact. Temperature scan at entry and exit points is not an efficient way to stop international spread since infected people may be within the incubation period and not show early symptoms during the disease. For an adequate risk assessment and a follow of a probable case, the patients should be provided with disease-preventing messages, health statements should be collected upon entry, and contact information of the passengers should be obtained, which would be much more efficient [18]. 

It is necessary to delay or prevent travel to affected regions for elderly patients and for those with underlying chronic diseases. Personal hygiene, coughing etiquette, and putting an at least one-meter distance between yourself and those showing symptoms are important for all passengers. Frequent hand hygiene after contact with particularly respiratory fluids is mandatory. Hand hygiene includes washing the hand with soap and water or cleaning the hand with alcohol-based liquids. The mouth and nose of the person coughing or sneezing should be closed with a handkerchief or using the inner part of the elbow. Touching the mouth and nose should be avoided [18]. 

Unless a person shows symptoms, s/he does not need to wear a medical mask since there is no evidence on any kind of masks protecting the noninfected person from the virus. Besides, masks can be commonly worn in some cultures. It is important to follow the best practice on how to wear, remove, and dispose of the mask, and ensure hand hygiene if medical masks are to be worn [18]. 

Passengers returning from the affected regions should monitor the symptoms for 14 days on their own and also follow the national protocols of the receiving countries. Some countries may demand the returning passengers to be put in quarantine. Should symptoms such as fever, cough, and difficulty in breathing manifest, it is recommended to the passengers to contact local healthcare providers preferably over the phone and inform them of their symptoms and travel history [18]. 

It is suggested to follow the recommendations of WHO for designated passengers in entry points. The management of ill passengers in the context of present COVID-19 disease pandemic in international airports, harbors, and motorway/road gates should include measures to be implemented according to the priorities and capacities of each country. In order to detect ill passengers and determine the symptoms of COVID-19 disease and the possibility of virus exposure, interviewing with ill passengers, reporting cases with suspected COVID-19 infection, isolation of those with suspected COVID-19 infection and initial case management and referral are necessary [20]. 

Airport operators, aircraft/airplane operators, airline and airport crew, and ground personnel should be informed on how to recognize COVID-19 signs and symptoms. Crew and ground personnel should be informed on and frequently reminded of measures preventing the spread of COVID-19 including social distancing, hand hygiene, respiratory etiquette, environmental cleaning, waste disposal, when and how to wear masks, and avoiding contact with people showing respiratory symptoms. Medical face mask should be reserved for individuals with respiratory symptoms to prevent contamination with others. The personnel should be trained for hand hygiene and how to wear and remove protective equipment. Personnel in close contact with symptomatic individuals should wear medical mask, eye protection (face shield or goggles), gloves, and gown [21]. 

A number of factors should be taken into account to prevent the spread of COVID-19 for countries that have decided to bring back citizens from affected regions. These include scanning right before flight, risk communication with the passengers and crew, infection control resources for the flight/journey, preparation of the crew for a possible infected passenger, entry scan upon arrival, and close-monitoring for 14 days after arrival [18]. 

Countries should intensify surveillance for severe pneumonia and uncommon flu-like disease pandemics and attentively monitor the development of COVID-19 pandemics by strengthening epidemiologic surveillance. Countries should continue raising awareness in the public opinion, healthcare specialists and policy-makers through effective risk communication regarding COVID-19 and should refrain from actions of stigmatization and discrimination. Countries should share all related information on COVID-19 for its timely evaluation and management as necessitated by the International Health Regulations (2005) [18]. 

Countries implementing additional health measures that significantly interfere with international traffic need to share related scientific data for the implementation of these measures and public health justification with WHO within the first 48 h after the implementation. WHO will share this information with other countries. Significant intervention generally means a more than 24-h delay in or rejection of international passengers, luggage, cargos, containers, vehicles, goods, and similar items [18]. Airline travel has the most important role in spreading pandemics. In a study considering airline network in the prediction of the spread of pandemics, rate and density of transmission have been detected with high accuracy [22]. Therefore, making predictions in early periods of pandemics using these modellings can be effective in rapidly taking necessary measures [23]. 

If a country decides to quarantine arriving passengers who do not show symptoms, some factors should be taken into consideration. There is no universal guideline regarding the infrastructure of the quarantine facility; however, an area that will not increase the potential contamination and those put in quarantine should be recorded to be followed in the event of potential disease. Accommodation and supplies, sufficient food and water to passengers, sleep arrangements and clothing, protection of luggage and other items, appropriate medical treatment, necessary communication devices should be adequately provided for in a language they can understand. Medical mask is not required for those put in quarantine. If masks are used, the best practice should be followed. Quarantine period, which lasts 14 days (according to the currently known incubation period of the virus), can be extended due to delayed exposure [24]. 

Social and economic life continues during pandemics. A pandemic has the potential to affect all sectors. In the event of declaration of disaster as regards the severity of the pandemic, there is legislation oriented at covering fiscal charges. However, regulation should be made for pandemics that do not necessitate declaration of disaster in order to meet unexpected/unanticipated needs, and additional financial needs should be met. During a rapidly spreading pandemic, vital setbacks can be seen in the transfer of goods and services. The need for social support programs due to economic problems arising from the shutdown of businesses and interim unemployment [25]. 

It is predicted that COVID-19 pandemic will cost the world economy as much as an approximate 1 trillion dollars, which is a much deeper and worse global crisis compared to that of 2007-2008. Institutions like the UN are run with the aid of developed countries in particular. It is feared that a serious crisis will be felt in UN resources as part of the effect of this pandemic and lead to weakness in the function of the UN [26]). 

Pandemic diseases may result in acute, short-term fiscal shocks and long-term damage in economic growth. Early period public health efforts (such as monitoring contact, implementing quarantine, isolating contagious cases) in order to cover or limit pandemics require significant human resources and personnel cost. As a pandemic expands, new facilities may be needed to be constructed to manage additional contagious cases, and health system expenses will tremendously increase as a result of demand in medical supplies, personal protective equipment, and medicine. Decreasing tax revenue may deepen the fiscal stress caused by increasing expenses in low- and middle-income countries that have weak tax systems and severe fiscal restrictions. This dynamic was seen in the Western Africa Ebola pandemic in Liberia in 2014. As costs increased, economic activity slowed down, and quarantines and curfews decreased the government’s capacity to collect revenues. During a mild-to-moderate pandemic, high income countries that are not affected can balance fiscal crises in low income countries by providing official recovery support including direct budget support. Meanwhile, high income countries may be faced with the same fiscal stresses and be unwilling to provide help. During a severe pandemic, low- and middle-income countries may cut back in government expenses. Negative economic crises are derived from workforce reduction due to disease and deaths and behavior change out of fear. Fear manifests itself with many behavioral changes. The analysis of the economic effects of the 2014 Western Africa Ebola pandemic has shown decrease in workforce, shutdown in businesses, delay in transportation, closing of land borders by some governments, restrictions implemented on citizens arriving from affected regions in entry to the country, cancellation of commercial flights, decrease in shipment and prevention in travel and trade. These effects decrease the participation of the pandemic to workforce and constrict local and regional trade. Preventive behavior (such as prevention of travel, restaurant and public spaces and workplace discontinuity as prophylactics) also has economic outcomes. During a severe pandemic, all sectors of the economy (agriculture, manufacture, services) encounter shortages, rapid price elevation is basic necessities, deterioration that causes economic stress for the household, private firms, and governments. A severe pandemic may result in a significant and permanent economic damage [27]. 

## 5. Conclusion

In order to take a pandemic under control, coordination should be established, flow of information should be regulated, necessary health interventions (case management algorithms, vector control) should be determined, health systems (hospitals, healthcare personnel, medicine) should be strengthened, the society should be informed, and the community should be included in pandemic surveillance and control.
